# Supplementary material for: Benzocarbazoledinones as SARS-CoV-2 Replication Inhibitors: Synthesis, Cell-Based Studies, Enzyme Inhibition, Molecular Modeling, and Pharmacokinetics Insights
Source: Viruses. 2024 Nov 13;16(11):1768. doi: 10.3390/v16111768 (PMC11598835; doi:10.3390/v16111768)

Supporting Information

Benzocarbazoledinones as SARS-CoV-2 Replication Inhibitors: Synthesis, Cell-based Studies, Enzyme Inhibition, and Molecular Modeling Insights

Luana G. de Souza‡, Eduarda A. Penna‡, Alice S. Rosa‡, Juliana C. da Silva, Edgar Schaeffer, Juliana V. Guimarães, Ellen A. Gomes, Dennis M. de Paiva, Vinicius C. de Souza, Vivian Neuza S. Ferreira, Daniel D. C. Souza, Sylvia Roxo, Giovanna B. Conceição, Larissa E. C. Constant, Stephany da S. Costa, Celimar Cinézia Silva, Diego Allonso, Priscila V. Z. Capriles Goliatt*,⸸, Milene D. Miranda*,⸸ and Alcides J. M. da Silva*,⸸

.

*Corresponding authors: A. J. M. da Silva (alcides@ippn.ufrj.com); Milene D. Miranda (mmiranda@ioc.fiocruz.br), Priscila V. Z. Capriles Goliatt (priscilacapriles@gmail.com);

‡These authors contributed equally to this work and shared the first authorship.

⸸These authors contributed equally to this work and shared the last authorship

TABLE OF CONTENTS

1. GENERAL EXPERIMENTAL PROCEDURES 4

2. SYNTHESIS OF THE STARTING MATERIALS 4

Procedure for the synthesis of 2-(benzylamino)naphthalene-1,4-dione. 4

Procedure for the synthesis of 2-aminonaphthalene-1,4-dione 4

3. SYNTHESIS OF THE INTERMEDIARIES 5

General procedure for the synthesis of 2-N-phenyl-aminonaphtoquinone. 5

2-(phenylamino)naphthalene-1,4-dione 5

2-((4-methoxyphenyl)amino)naphthalene-1,4-dione 5

2-(p-tolylamino)naphthalene-1,4-dione 5

2-((2-methoxyphenyl)amino)naphthalene-1,4-dione 6

2-((2,4-dimethoxyphenyl)amino)naphthalene-1,4-dione 6

ethyl 4-((1,4-dioxo-1,4-dihydronaphthalen-2-yl)amino)benzoate 6

2-((4-fluorophenyl)amino)naphthalene-1,4-dione 6

2-(pyridin-2-ylamino)naphthalene-1,4-dione 7

2-(naphthalen-1-ylamino)naphthalene-1,4-dione 7

4. COMPUTATIONAL STUDIES 8

5. REFERENCES 9

6. NMR SPECTRA OF THE NOVEL 2-N-PHENYL-AMINONAPHTOQUINONE: 6f and 6j 10

^{1}H NMR (400 MHz, $CDCl_3$) spectrum of ethyl 4-((1,4-dioxo-1,4-dihydronaphthalen-2-yl)amino)benzoate (6f) 10

^{13}C{H} NMR (101 MHz, $CDCl_3$) spectrum of ethyl 4-((1,4-dioxo-1,4-dihydronaphthalen-2-yl)amino)benzoate (6f) 10

^{1}H NMR (500 MHz, $CDCl_3$) spectrum of 2-(pyridin-2-ylamino)naphthalene-1,4-dione (6j) 11

^{13}C{H} NMR (126 MHz, $CDCl_3$) spectrum of 2-(pyridin-2-ylamino)naphthalene-1,4-dione (6j) 11

7. NMR SPECTRA OF THE BENZOCARBAZOLEDINONES 12

^{1}H NMR (500 MHz, DMSO-d_6) spectrum of 5H-benzo[b]carbazole-6,11-dione 12

$^{13}C\{H\}$ NMR (126 MHz, DMSO-d_6) spectrum of 5H-benzo[b]carbazole-6,11-dione 12

^{1}H NMR (500 MHz, $CDCl_3$) spectrum of 2-methoxy-5H-benzo[b]carbazole-6,11-dione 13

$^{13}C\{H\}$ NMR (126 MHz, DMSO-d_6) spectrum of 2-methoxy-5H-benzo[b]carbazole-6,11-dione 13

^{1}H NMR (500 MHz, $CDCl_3$) spectrum of 2-methyl-5H-benzo[b]carbazole-6,11-dione 14

$^{13}C\{H\}$ NMR (126 MHz, $CDCl_3$) spectrum of 2-methyl-5H-benzo[b]carbazole-6,11-dione 14

^{1}H NMR (500 MHz, $CDCl_3$) spectrum of 4-methoxy-5H-benzo[b]carbazole-6,11-dione 15

$^{13}C\{H\}$ NMR (126 MHz, $CDCl_3$) spectrum of 4-methoxy-5H-benzo[b]carbazole-6,11-dione 15

^{1}H NMR (500 MHz, $CDCl_3$) spectrum of 2,4-dimethoxy-5H-benzo[b]carbazole-6,11-dione 16

$^{13}C\{H\}$ NMR (126 MHz, $CDCl_3$) spectrum of 2,4-dimethoxy-5H-benzo[b]carbazole-6,11-dione 16

^{1}H NMR (500 MHz, $CDCl_3$) spectrum of ethyl 6,11-dioxo-6,11-dihydro-5H-benzo[b]carbazole-2-carboxylate 17

$^{13}C\{H\}$ NMR (126 MHz, $CDCl_3$) spectrum of ethyl 6,11-dioxo-6,11-dihydro-5H-benzo[b]carbazole-2-carboxylate 17

^{1}H NMR (400 MHz, $(CD_3)_2CO$) spectrum of 2-fluoro-5H-benzo[b]carbazole-6,11-dione 18

$^{13}C\{H\}$ NMR (101 MHz, $(CD_3)_2CO$) spectrum of 2-fluoro-5H-benzo[b]carbazole-6,11-dione 18

^{1}H NMR (500 MHz, $CDCl_3$) spectrum of 7H-dibenzo[a,h]carbazole-7,12(13H)-dione 19

$^{13}C\{H\}$ NMR (126 MHz, $CDCl_3$) spectrum of 7H-dibenzo[a,h]carbazole-7,12(13H)-dione 19

1. GENERAL EXPERIMENTAL PROCEDURES

All commercial reagents and solvents were purchased from Sigma-Aldrich, TCI, Oakwood Chemical, or Acros Organics and used without further purification. All reactions that required heating were performed using an oil-bath. Analytical thin layer chromatography (TLC) was performed on 0.25 mm silica gel 60 F_{254} plates and visualized under UV light (254 nm or 365 nm) or by staining with vanillin/H_2SO_4. Preparative TLC was performed on 20 x 20 cm glass backed plates bearing a 0.5 mm layer of silica gel 60 F_{254} (15-40 μm). Flash column chromatography was performed on silica gel 60 (230-400 mesh) SiliaFlash^{TM}. NMR spectra were recorded on Varian Unity 400 or 500 MHz instruments at 25 °C. Chemical shifts are expressed in ppm relative to TMS (Me_4Si) or deuterated solvent ($CDCl_3$, DMSO-*d6*) and the coupling constants are expressed in Hz. High-resolution mass spectra (HRMS) were obtained with a Solarix XR mass spectrometer with Electrospray Ionization (ESI) source coupled to Fourier Transform-Ion Cyclotron Resonance (FT-ICR) mass analyzer.

2. SYNTHESIS OF THE STARTING MATERIALS

Procedure for the synthesis of 2-(benzylamino)naphthalene-1,4-dione. In a pyrex tube were added the 2-bromo-1,4-naphthoquinone (2.11 mmol, 0.5 g, 1 equiv), the benzylamine (4.22 mmol, 0.4515 g, 2 equiv) and K_2CO_3 (5.25 mmol, 0.755 g, 2.5 equiv) in *t*-BuOH (15 mL) for 24h under stirring and heating at 80ºC. The mixture was diluted in AcOEt and washed with NH_4Cl (5 x 25 mL) dried over sodium sulfate and concentrated under reduced pressure. The solid was recrystallization with ethanol, yielding the pure product as a orange solid (0.511 g, 92% yield). **^{1}H NMR** (500 MHz, $CDCl_3$) δ 8.09 (dd, 2H), 7.74 (t, 1H), 7.64 (t, 1H), 7.35 (m, 5H), 6.20 (s largo, 1H), 5.79 (s, 1H), 4.38 (d, 2H) ppm. The spectroscopic data were consistent with those reported in the literature.[27]

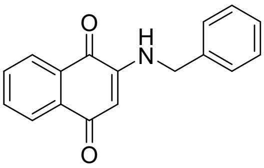

Procedure for the synthesis of 2-aminonaphthalene-1,4-dione. In a pyrex tube, 2-(benzylamino)naphthalene-1,4-dione (2.0 mmol, 0.526 g, 1 equiv) and Pd/C 10% (0.6 mmol, 0.638 g, 30 mol%) were added in 10 mL of THF:Isopropanol (3:1) under H_2 1 atm for 2.5h in room temperature. The mixture was diluted in AcOEt, filtered in celite and and concentrated under reduced pressure. The product was obtained with quantitative yield (0.343 g, ~100%) as a brown solid. **^{1}H NMR** (500 MHz, $CDCl_3$) δ 8.07 (dd, 2H), 7.68 (m, 2H), 6.00 (s, 1H), 5.18 (s, 2H) ppm. The spectroscopic data were consistent with those reported in the literature.[28]

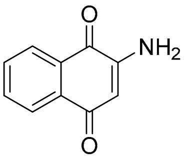

3. SYNTHESIS OF THE INTERMEDIARIES

General procedure for the synthesis of 2-N-phenyl-aminonaphtoquinone. In a pyrex tube were added a suspension of $Pd(OAc)_2$ (0.0028 g, 0.01 mmol), XPhos (0.0012 g, 0.02 mmol), NaOtBu (0.034 g, 0.35 mmol), aryl halite (0.25 mmol) and 2-aminoquinone (**4**) (0.0519 g, 0.3 mmol) in toluene and heated under nitrogen atmosphere and microwave irradiation (150 ºC, between 3 to 9h, infrared probe). Then, the mixture was allowed to cool to rt, diluted in AcOEt, filtered in celite, and concentrated under reduced pressure. The crude material was purified by a silica gel column with dichloromethane as solvent.

2-(phenylamino)naphthalene-1,4-dione

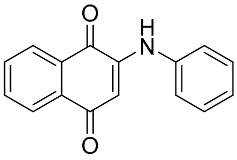

The compound was obtained as a red solid (0.048 g, 77% yield). **^{1}H NMR** (500 MHz, $CDCl_3$) δ 8.12 (td, 2H), 7.77 (td, 1H), 7.68 (td, 1H), 7.57 (s largo, 1H), 7.43 (m, 2H), 7.28 (m, 2H), 7.23 (t, 1H), 6,42 (s, 1H) ppm. The spectroscopic data were consistent with those reported in the literature.[29]

2-((4-methoxyphenyl)amino)naphthalene-1,4-dione

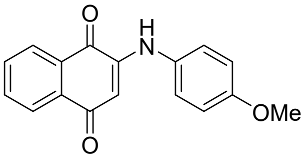

The compound was obtained as a red solid (0.049 g, 70% yield). **^{1}H NMR** (500 MHz, $CDCl_3$) δ 8.10 (ddd, J = 7.5, 4.6, 1.3 Hz, 2H), 7.75 (td, J = 7.6, 1.3 Hz, 1H), 7.65 (td, J = 7.6, 1.3 Hz, 1H), 7.44 (s, 1H), 7.23 – 7.16 (m, 2H), 7.01 – 6.90 (m, 2H), 6.22 (s, 1H), 3.83 (s, 3H). The spectroscopic data were consistent with those reported in the literature.[29]

2-(p-tolylamino)naphthalene-1,4-dione

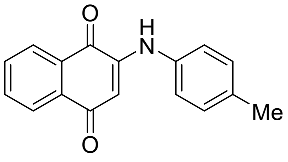

The compound was obtained as a red solid (0.052 g, 79% yield). **^{1}H NMR** (500 MHz, $CDCl_3$) δ 8.11 (ddd, J = 7.3, 5.6, 1.3 Hz, 2H), 7.75 (td, J = 7.6, 1.3 Hz, 1H), 7.66 (td, J = 7.5, 1.3 Hz, 1H), 7.51 (s, 1H), 7.23 – 7.15 (m, 4H), 6.35 (s, 1H), 2.37 (s, 3H). The spectroscopic data were consistent with those reported in the literature.[29]

2-((2-methoxyphenyl)amino)naphthalene-1,4-dione

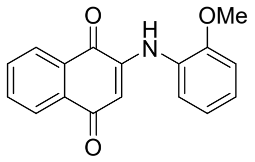

The compound was obtained as a red solid (0.045 g, 65% yield). **^{1}H NMR** (500 MHz, $CDCl_3$) δ 8.12 (td, J = 7.5, 1.3 Hz, 2H), 7.99 (s, 1H), 7.76 (td, J = 7.5, 1.4 Hz, 1H), 7.67 (td, J = 7.5, 1.4 Hz, 1H), 7.43 (dd, J = 7.9, 1.6 Hz, 1H), 7.15 (td, J = 7.9, 1.6 Hz, 1H), 7.06 – 6.94 (m, 2H), 6.49 (s, 1H), 3.92 (s, 3H). The spectroscopic data were consistent with those reported in the literature.[30]

2-((2,4-dimethoxyphenyl)amino)naphthalene-1,4-dione

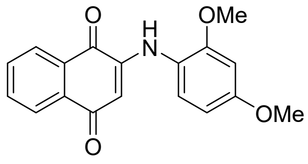

The compound was obtained as a red solid (0.053 g, 69% yield). **^{1}H NMR** (500 MHz, $CDCl_3$) δ 8.10 (ddd, J = 7.5, 2.7, 1.3 Hz, 2H), 7.75 (td, J = 7.6, 1.4 Hz, 1H), 7.66 (td, J = 7.5, 1.3 Hz, 1H), 7.52 (s, 1H), 6.47 (s, 1H), 6.41 (d, J = 2.1 Hz, 2H), 6.30 (t, J = 2.2 Hz, 1H), 3.80 (s, 6H). The spectroscopic data were consistent with those reported in the literature.[29]

ethyl 4-((1,4-dioxo-1,4-dihydronaphthalen-2-yl)amino)benzoate

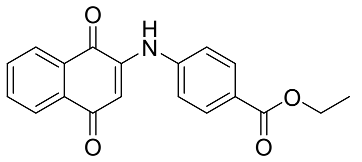

The compound was obtained as a red solid (0.048 g, 60% yield). **^{1}H NMR** (500 MHz, $CDCl_3$) δ 8.13 (ddd, J = 9.0, 7.7, 1.4 Hz, 2H), 8.11 – 8.08 (m, 2H), 7.78 (td, J = 7.5, 1.4 Hz, 1H), 7.74 (s, 1H), 7.70 (td, J = 7.6, 1.3 Hz, 1H), 7.35 – 7.30 (m, 2H), 6.58 (s, 1H), 4.39 (q, J = 7.2 Hz, 2H), 1.41 (t, J = 7.1 Hz, 3H). **^{13}C NMR** (101 MHz, $CDCl_3$) δ 184.07, 181.76, 165.78, 143.43, 141.80, 135.10, 132.93, 132.68, 131.30, 130.21, 126.71, 126.27, 120.80, 116.83, 105.19, 61.12, 14.34.

2-((4-fluorophenyl)amino)naphthalene-1,4-dione

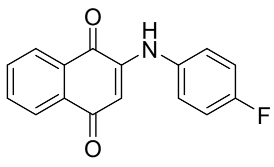

The compound was obtained as a red solid (0.027 g, 41% yield). **^{1}H NMR** (500 MHz, DMSO-d_6) δ 9.24 (s, 1H), 8.06 (dd, J = 7.7, 1.3 Hz, 1H), 7.95 (dd, J = 7.7, 1.3 Hz, 1H), 7.86 (td, J = 7.5, 1.4 Hz, 1H), 7.79 (td, J = 7.5, 1.4 Hz, 1H), 7.45 – 7.38 (m, 2H), 7.33 – 7.24 (m, 2H), 6.00 (s, 1H). The spectroscopic data were consistent with those reported in the literature.[29]

2-(pyridin-2-ylamino)naphthalene-1,4-dione

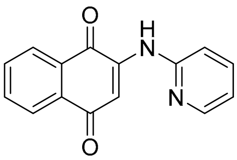

The compound was obtained as a red solid (0.046 g, 73% yield). ^{1}**H NMR** (500 MHz, $CDCl_3$) δ 8.41 (dd, J = 5.3, 1.9 Hz, 1H), 8.13 (dt, J = 7.6, 1.6 Hz, 2H), 8.05 (s, 1H), 7.99 (s, 1H), 7.77 (td, J = 7.5, 1.4 Hz, 1H), 7.73 – 7.62 (m, 2H), 7.03 – 6.95 (m, 2H). ^{13}**C NMR** (101 MHz, $CDCl_3$) δ 185.12, 182.01, 152.84, 148.33, 141.67, 137.82, 134.84, 132.86, 132.57, 130.27, 126.61, 126.19, 118.16, 113.55, 110.72, 110.00.

2-(naphthalen-1-ylamino)naphthalene-1,4-dione

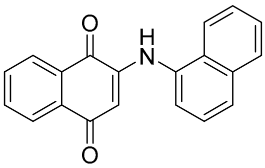

The compound was obtained as a red solid (0.05 g, 67% yield). ^{1}**H NMR** (500 MHz, $CDCl_3$) δ 8.17 (dd, J = 7.6, 1.3 Hz, 1H), 8.11 – 8.08 (m, 1H), 7.91 (ddt, J = 10.1, 7.6, 3.5 Hz, 2H), 7.82 (d, J = 7.9 Hz, 1H), 7.79 (s, 1H), 7.76 (td, J = 7.5, 1.3 Hz, 1H), 7.69 (td, J = 7.6, 1.2 Hz, 1H), 7.57 – 7.50 (m, 4H), 6.01 (s, 1H). The spectroscopic data were consistent with those reported in the literature.[30]

4. COMPUTATIONAL STUDIES

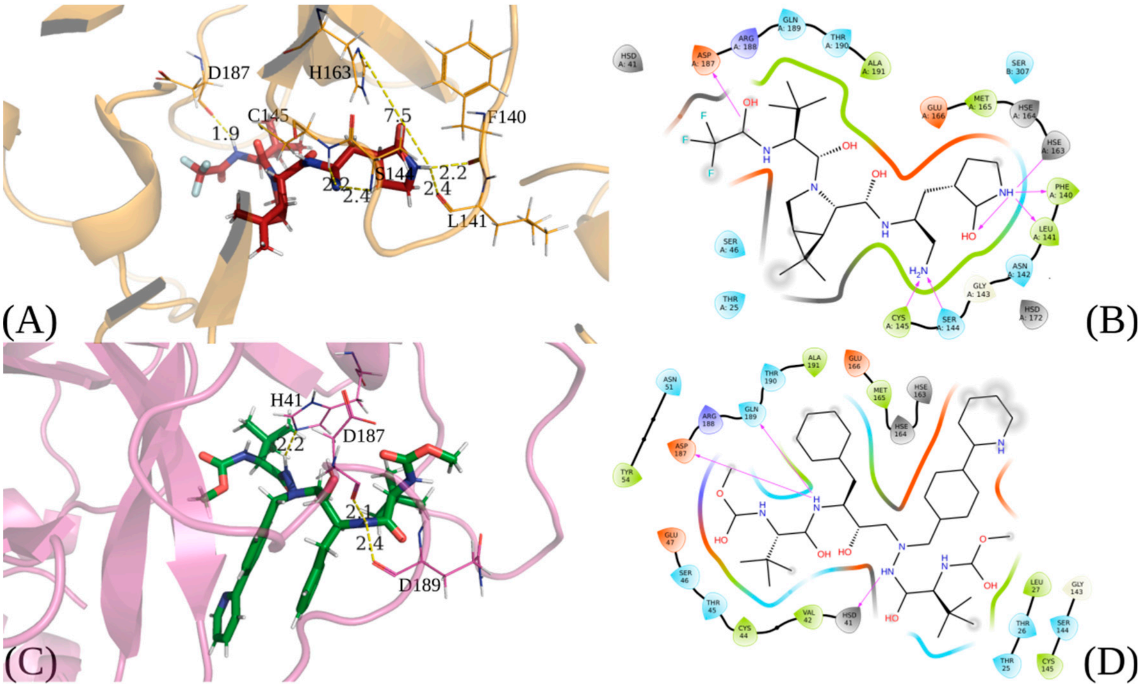

Figure S1. These results were obtained from docking with chain A of the 6209 Mpro protein. The representations were created in both three-dimensional and two-dimensional models to illustrate the interactions between the amino acid residues and the compounds, as well as their pharmacophoric profiles. The Mpro structure is depicted in cartoon form: Light Orange (Nirmatrelvir) and Purple (Atazanavir). The compounds are represented as sticks: Nirmatrelvir (dark red) and Atazanavir (dark green). Figures "A" and "B" represent Nirmatrelvir, while figures "C" and "D" represent Atazanavir, both illustrating their specific chemical interactions. In figures "B" and "D," the purple arrows highlight hydrogen bonds. The images were generated using PyMOL, version 2.1.0, and Maestro, version 13.6.122, Schrödinger - LLC.

Table S1. Theoretical physicochemical properties of benzocarbazoledinones **4a** and **4b** calculated using Swiss-ADME (SA) and Molinspiration (MI) servers.

Compounds	HBD		HBA		nRotB		MW (g/mol)		cLogP w/o		TPSA (Å²)		Unbound fraction
	SA	MI	SA	MI	SA	MI	SA	MI	SA	MI	SA	MI	DP
**4a**	1	1	2	3	0	0	247.25	247.25	2.76	3.55	49.93	49.93	42.41%
**4b**	1	1	3	4	1	1	277.27	277.28	2.75	3.58	59.16	59.17	36.65%
**ATV**	5	5	9	13	22	18	704.86	704.87	3.82	7.97	171.22	171.22	12.96%
**NMV**	3	3	8	9	11	8	499.53	499.53	1.64	1.66	131.40	131.40	23.60%

HBD: hydrogen bond donor; HBA: hydrogen bond acceptor; nRotB: number of rotatable bonds; MW: molecular weight; cLogP w/o: calculated logarithm of partition coefficient between *n*-octanol and water; TPSA: topological polar surface area. The values marked in bold indicate violations of Lipinski and Veber criteria. ATV: Atazanavir. NMV: Nirmatrelvir

5. REFERENCES

(27) B. J. Josey, E. S. Inks, X. Wen, C. J. Chou, Structure−Activity Relationship Study of Vitamin K Derivatives Yields Highly Potent Neuroprotective Agents. *J. Med. Chem.* **2013**, 56, 1007-1022. dx.doi.org/10.1021/jm301485d

(28) F. C. Demidoff, E. J. P. Rodrigues-Filho, A. L. F. de Souza, C. D. Netto, L. L. de Carvalho, Cross-Coupling Reactions with 2-Amino-/Acetylamino-Substituted 3-Iodo-1,4-naphthoquinones: Convenient Synthesis of Novel Alkenyl- and Alkynylnaphthoquinones and Derivatives. *Synthesis* **2021**, 53, 4097-4109. DOI: 10.1055/s-0037-1610781.

(29) X.-L. Chen, Y. Dong, S. He, R. Zhang, H. Zhang, L. Tang, X.-M. Zhang, J.-Y. Wang, A One-Pot Approach to 2-(N-Substituted Amino)-1,4-naphthoquinones with Use of Nitro Compounds and 1,4-Naphthoquinones in Water. *Synlett* **2019**, 30, 615-619. DOI: 10.1055/s-0037-1610689

(30) I. Sieveking, P. Thomas, J. C. Estévez, N. Quiñones, M. A. Cuéllar, J. Villena, C. Espinosa-Bustos, A. Fierro, R. A. Tapia, J. D. Maya, R. López-Muñoz, B. K. Cassels, R. J. Estévez, C. O. Salas, 2-Phenylaminonaphthoquinones and related compounds: Synthesis, trypanocidal and cytotoxic activities, *Bioorg. Med. Chem.* **2014**, 22, 4609–4620. http://dx.doi.org/10.1016/j.bmc.2014.07.030

6. NMR SPECTRA OF THE NOVEL 2-N-PHENYL-AMINONAPHTOQUINONE: 6f and 6j

^{1}H NMR (400 MHz, CDCl$_3$) spectrum of ethyl 4-((1,4-dioxo-1,4-dihydronaphthalen-2-yl)amino)benzoate (6f)

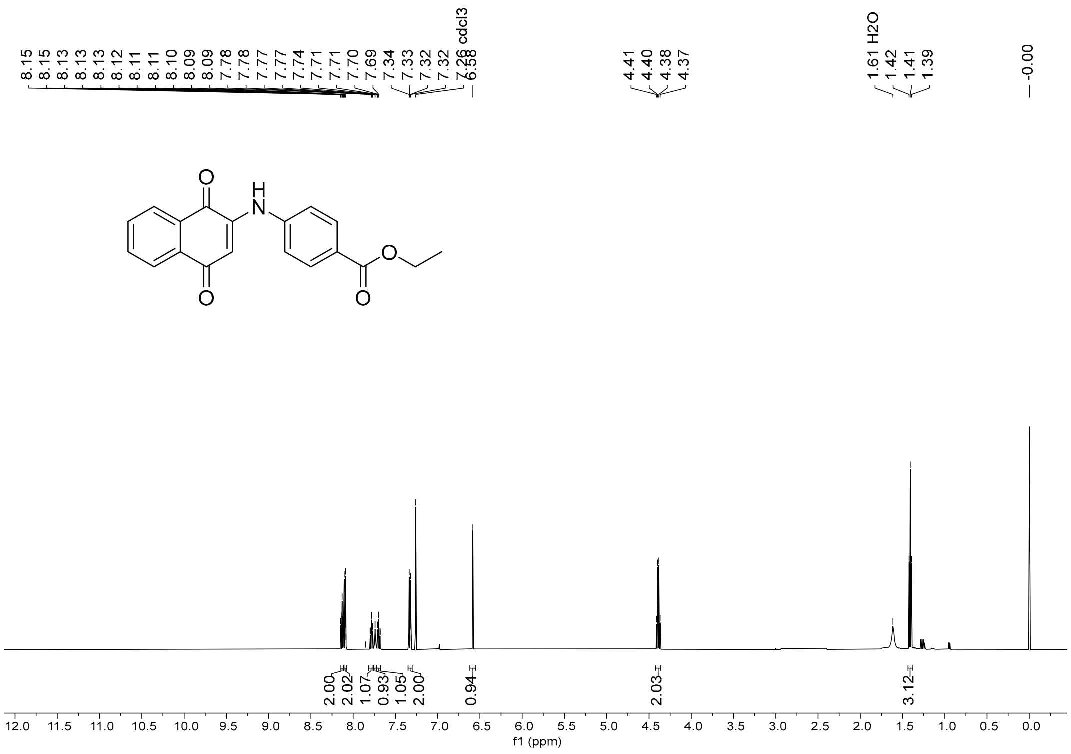

^{13}C{H} NMR (101 MHz, CDCl$_3$) spectrum of ethyl 4-((1,4-dioxo-1,4-dihydronaphthalen-2-yl)amino)benzoate (6f)

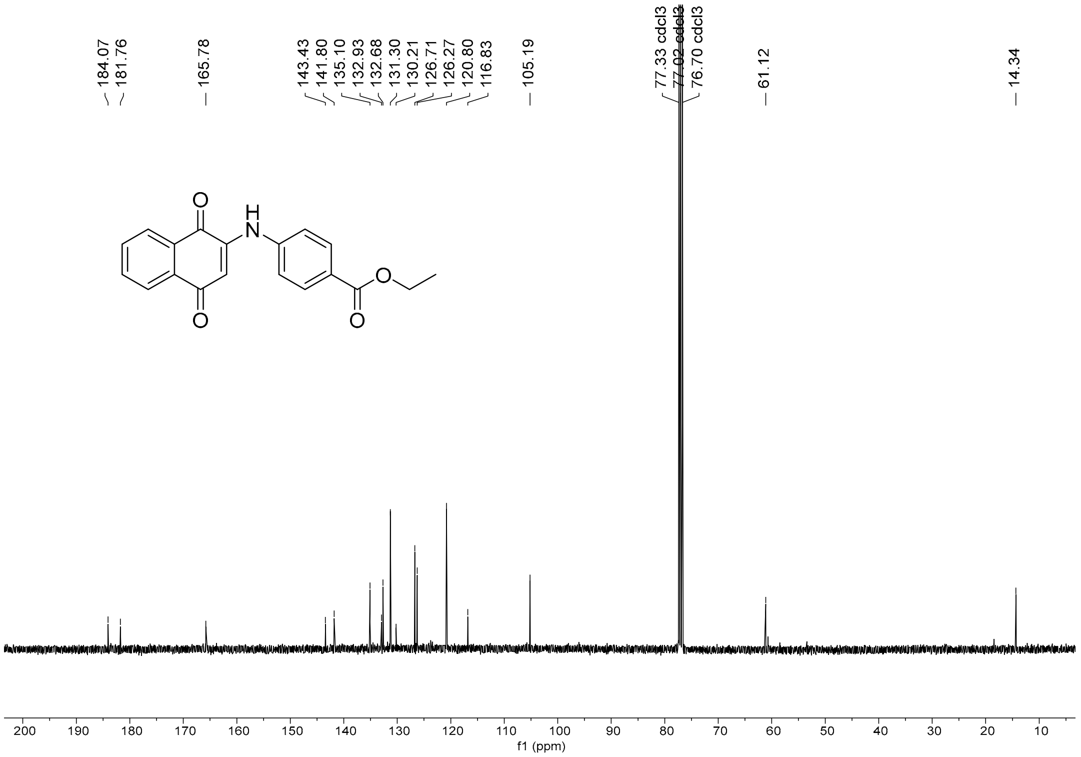

^{1}H NMR (500 MHz, $CDCl_3$) spectrum of 2-(pyridin-2-ylamino)naphthalene-1,4-dione (6j)

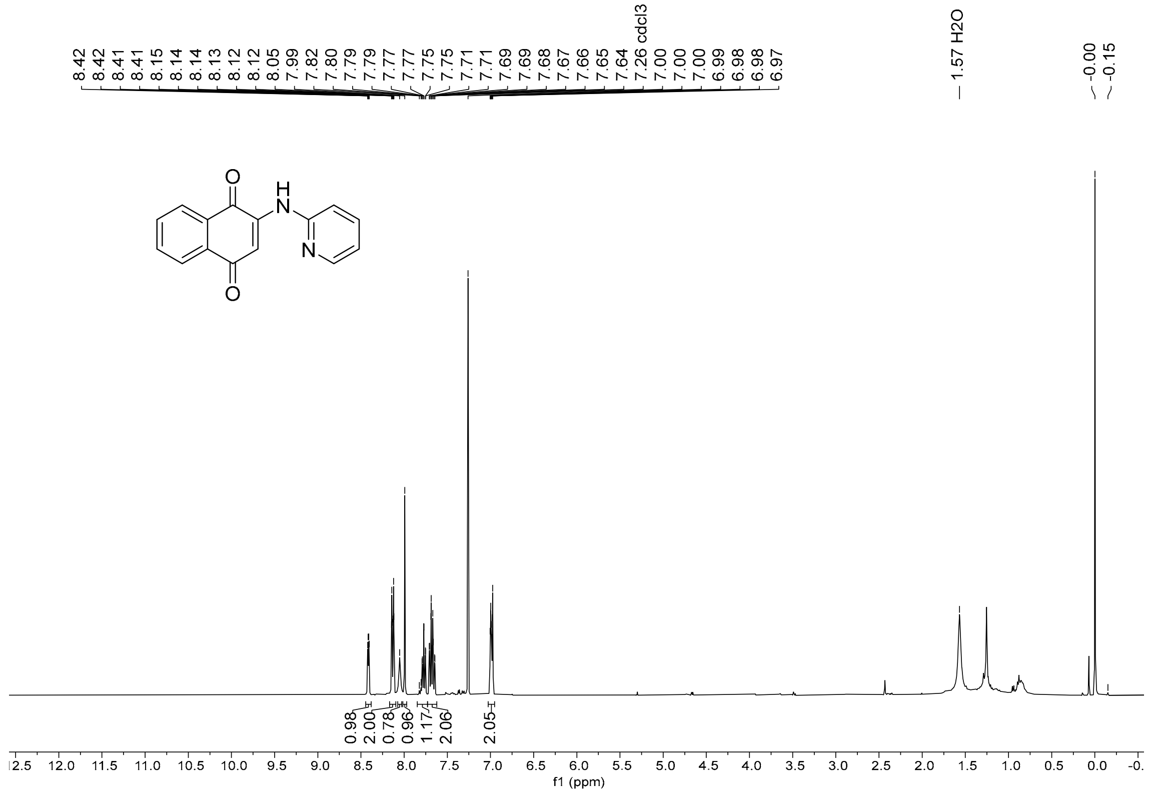

$^{13}C\{H\}$ NMR (126 MHz, $CDCl_3$) spectrum of 2-(pyridin-2-ylamino)naphthalene-1,4-dione (6j)

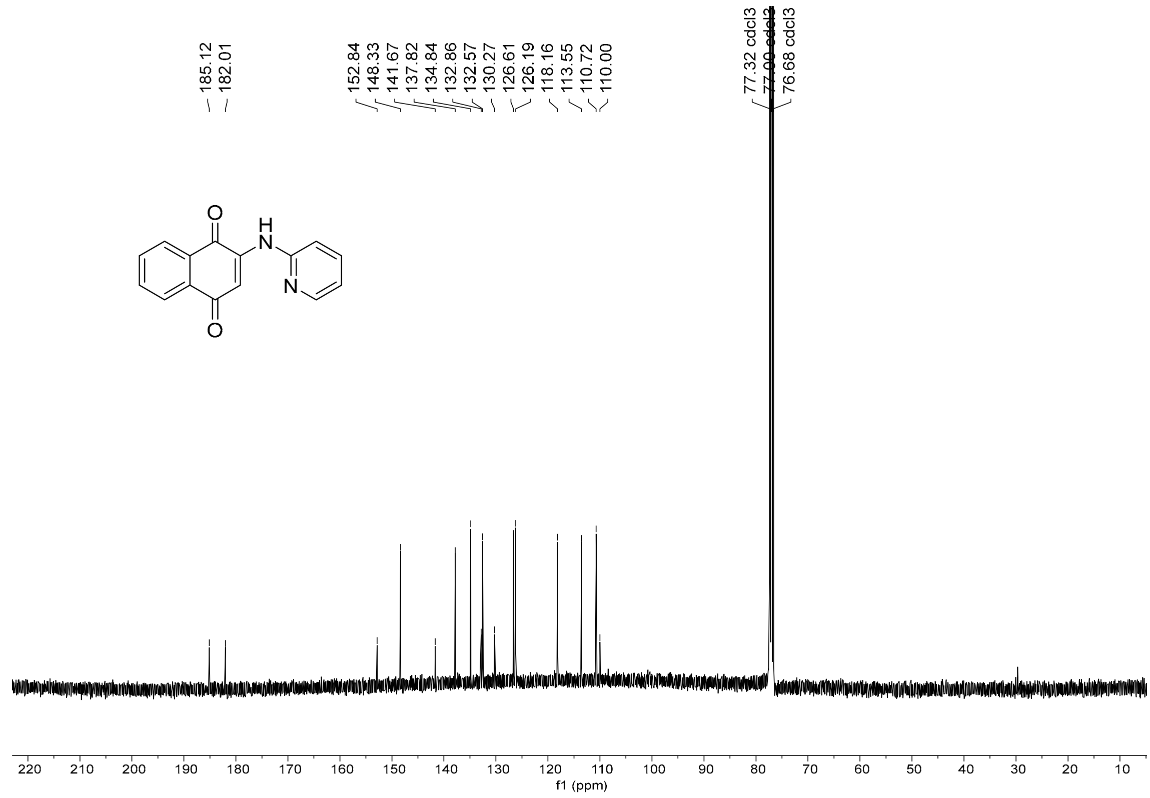

7. NMR SPECTRA OF THE BENZOCARBAZOLEDINONES

^{1}H NMR (500 MHz, DMSO-d_6) spectrum of 5H-benzo[b]carbazole-6,11-dione

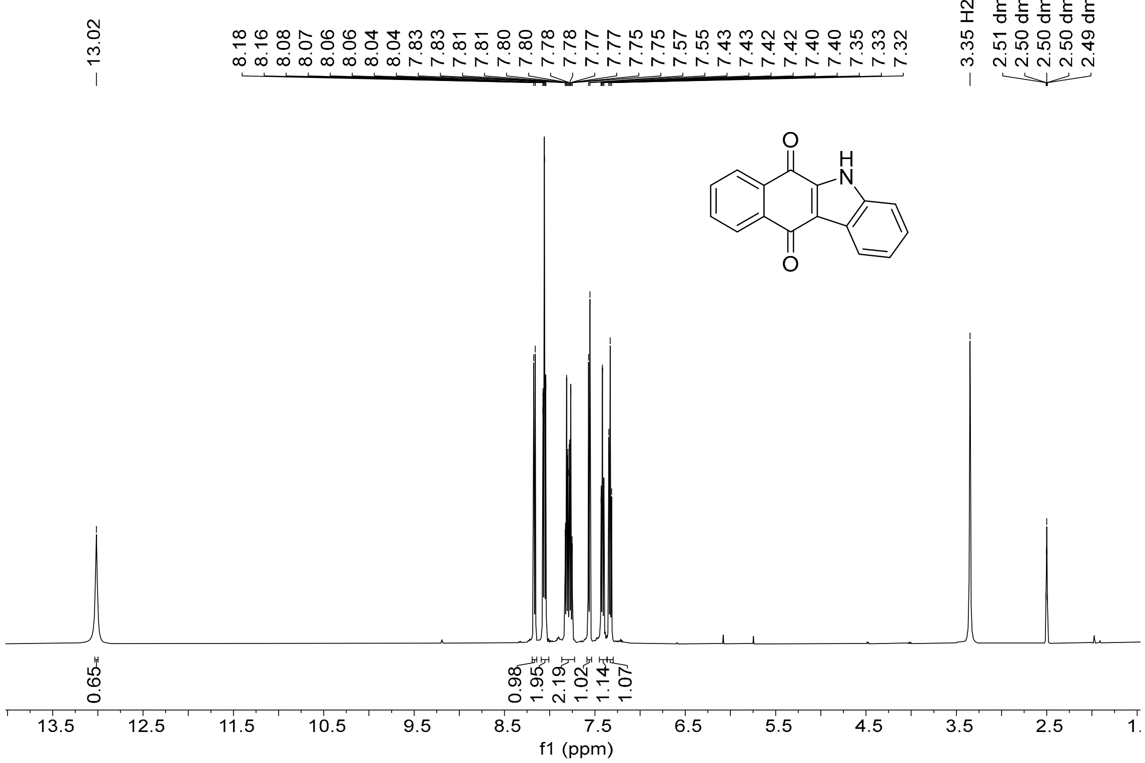

^{13}C{H} NMR (126 MHz, DMSO-d_6) spectrum of 5H-benzo[b]carbazole-6,11-dione

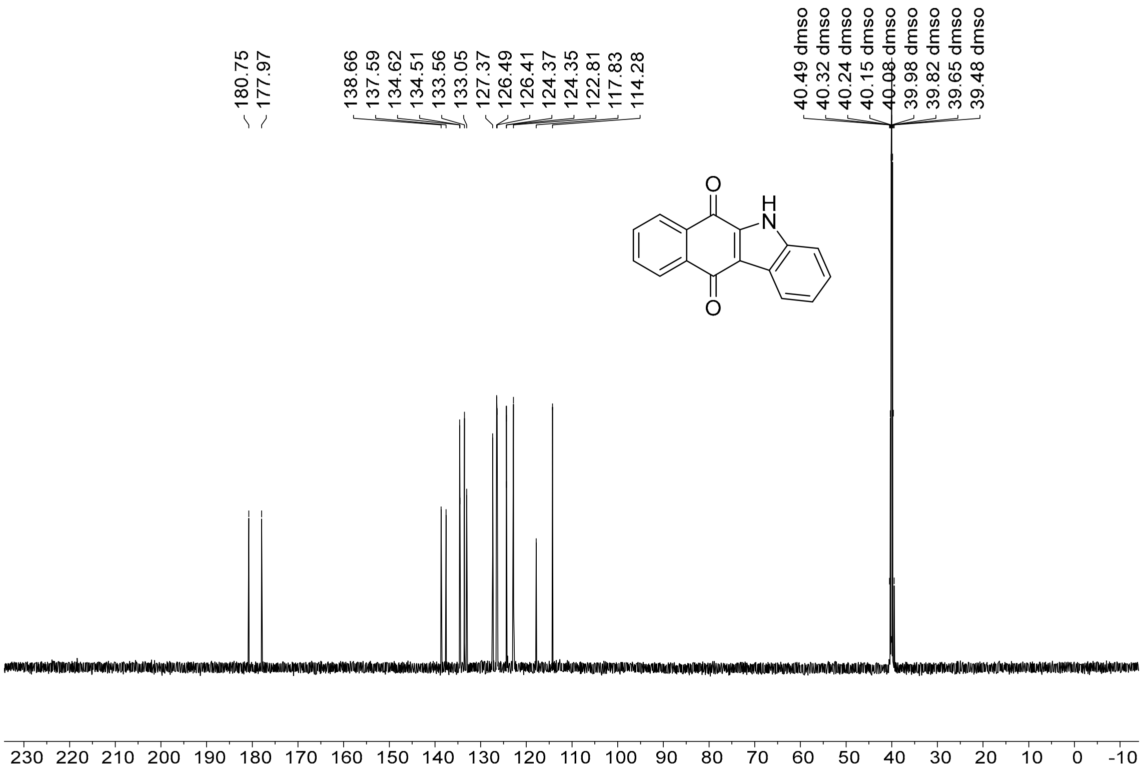

^{1}H NMR (500 MHz, CDCl$_3$) spectrum of 2-methoxy-5H-benzo[b]carbazole-6,11-dione

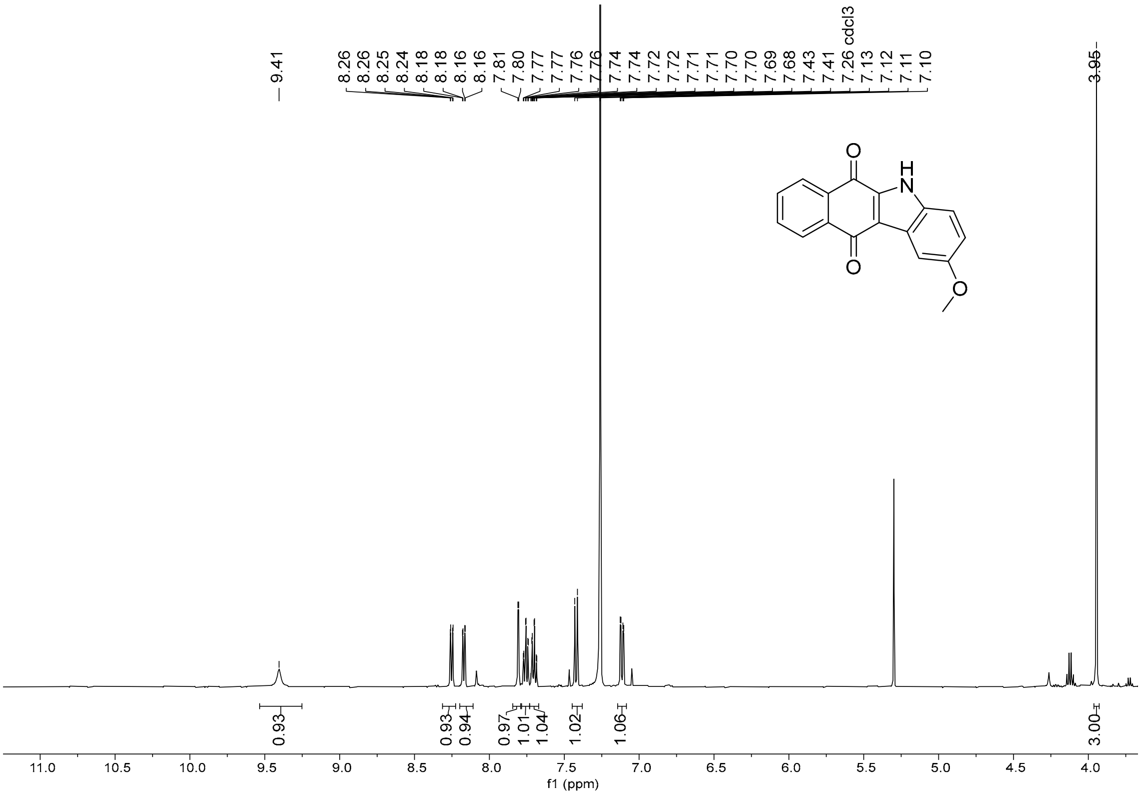

^{13}C{H} NMR (126 MHz, DMSO-d_6) spectrum of 2-methoxy-5H-benzo[b]carbazole-6,11-dione

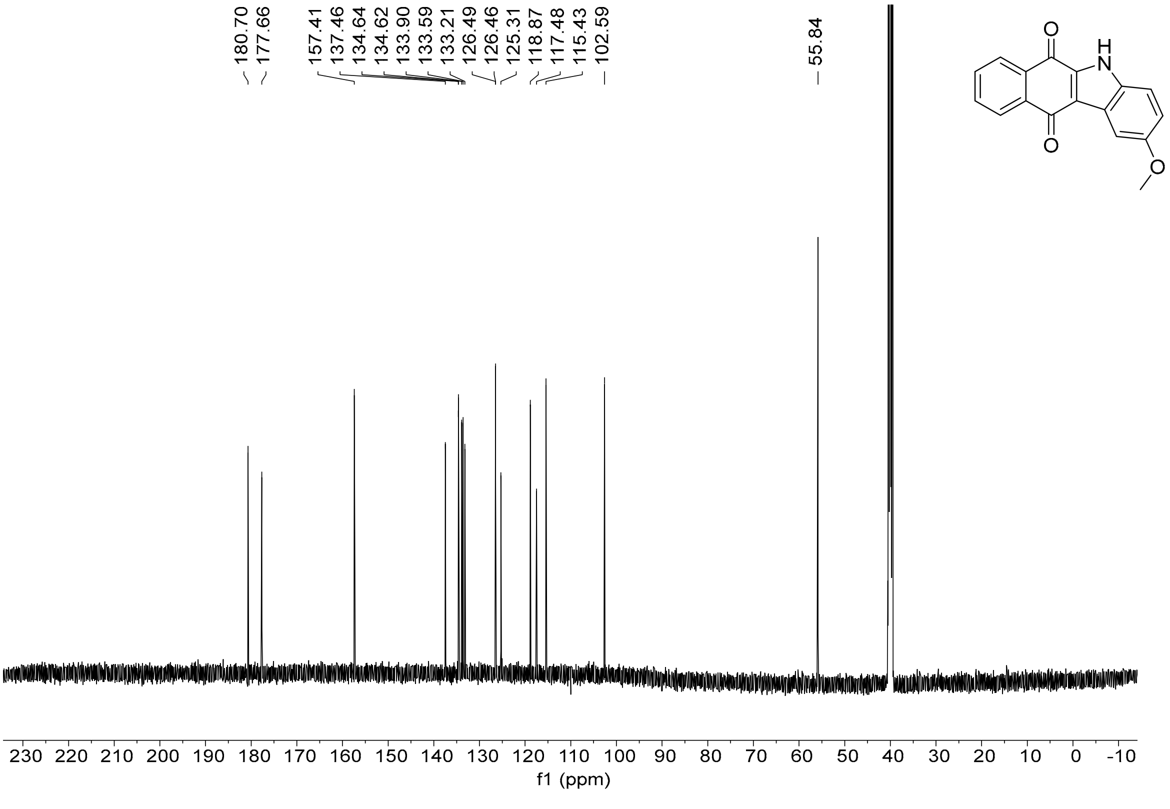

^{1}H NMR (500 MHz, $CDCl_3$) spectrum of 2-methyl-5H-benzo[b]carbazole-6,11-dione

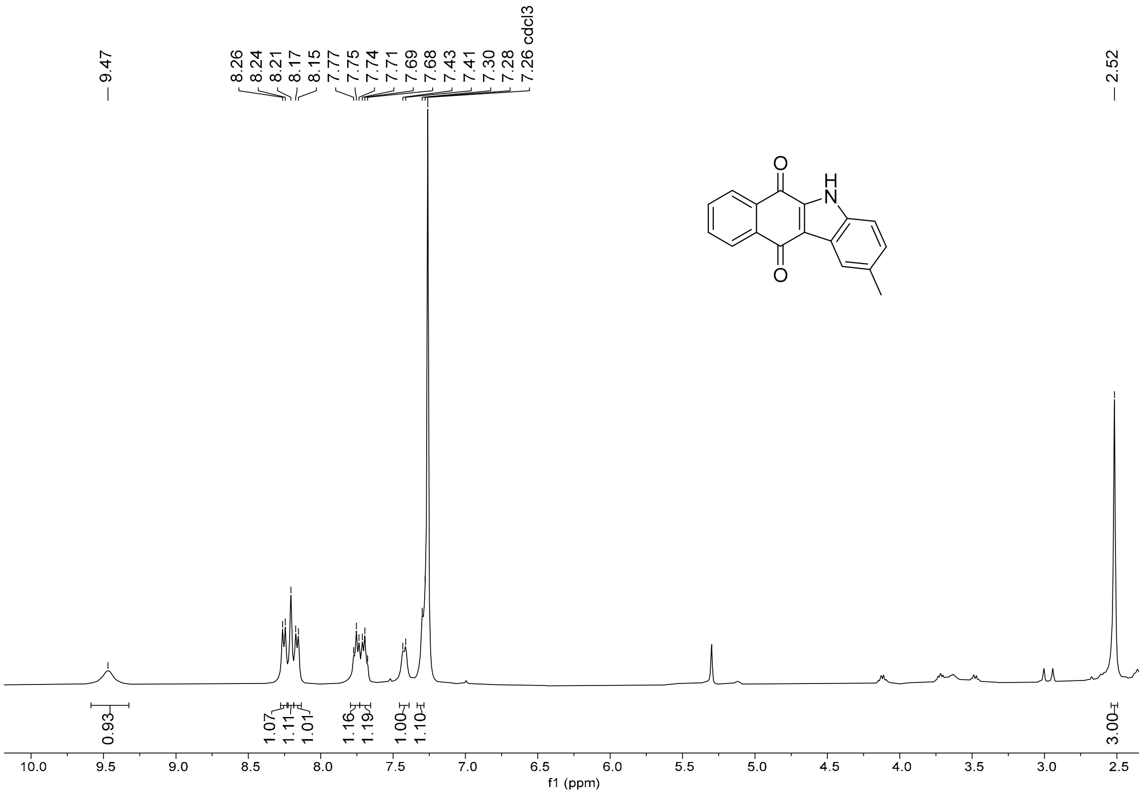

^{13}C{H} NMR (126 MHz, $CDCl_3$) spectrum of 2-methyl-5H-benzo[b]carbazole-6,11-dione

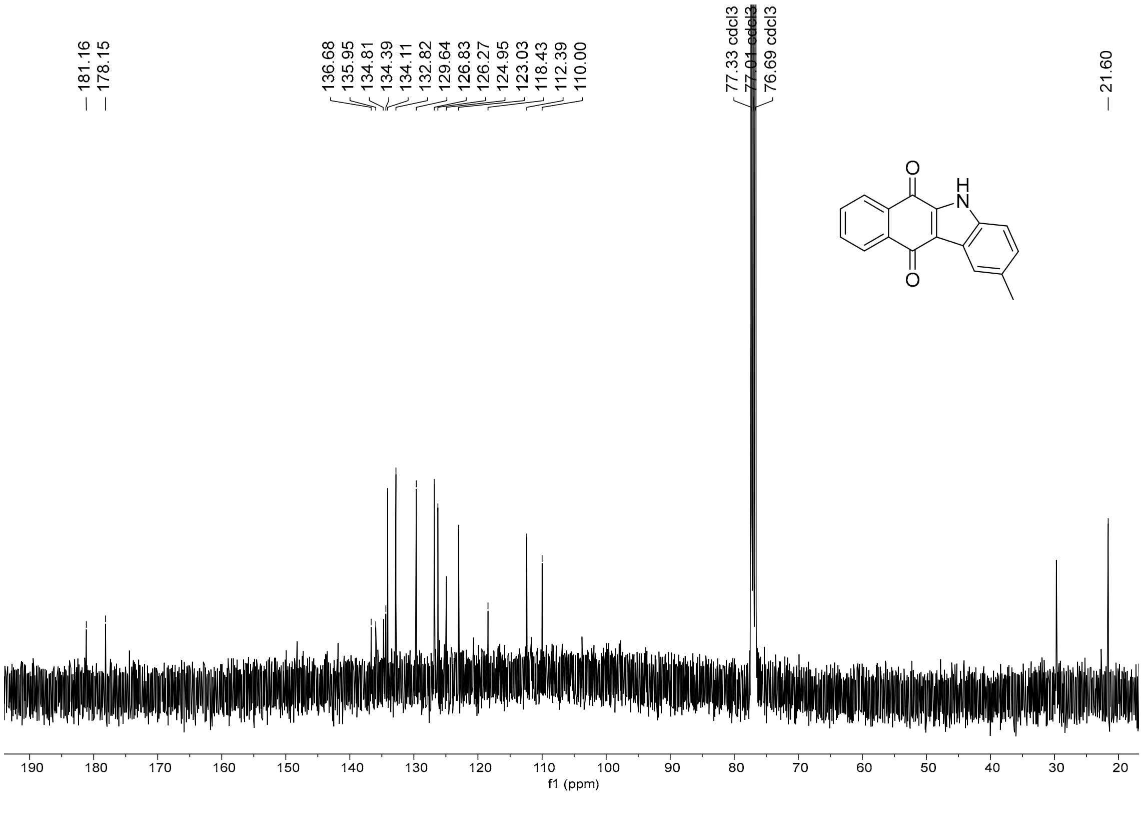

^{1}H NMR (500 MHz, CDCl$_3$) spectrum of 4-methoxy-5H-benzo[b]carbazole-6,11-dione

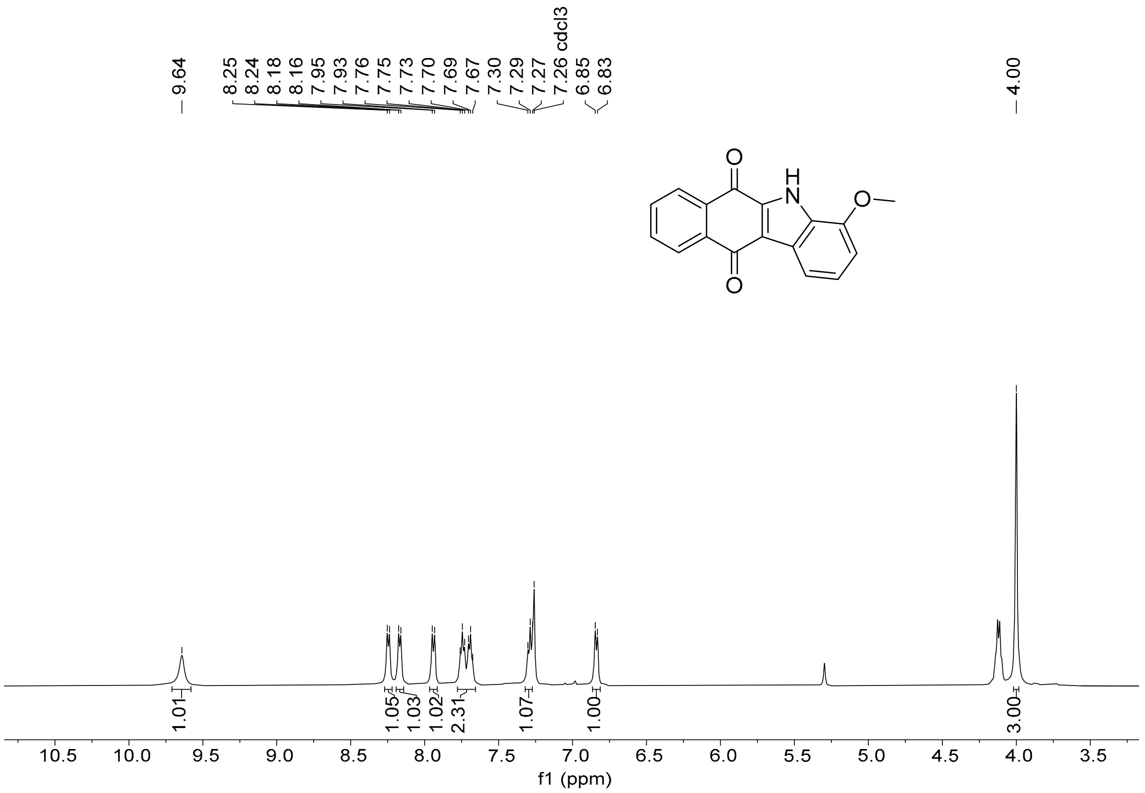

^{13}C{H} NMR (126 MHz, CDCl$_3$) spectrum of 4-methoxy-5H-benzo[b]carbazole-6,11-dione

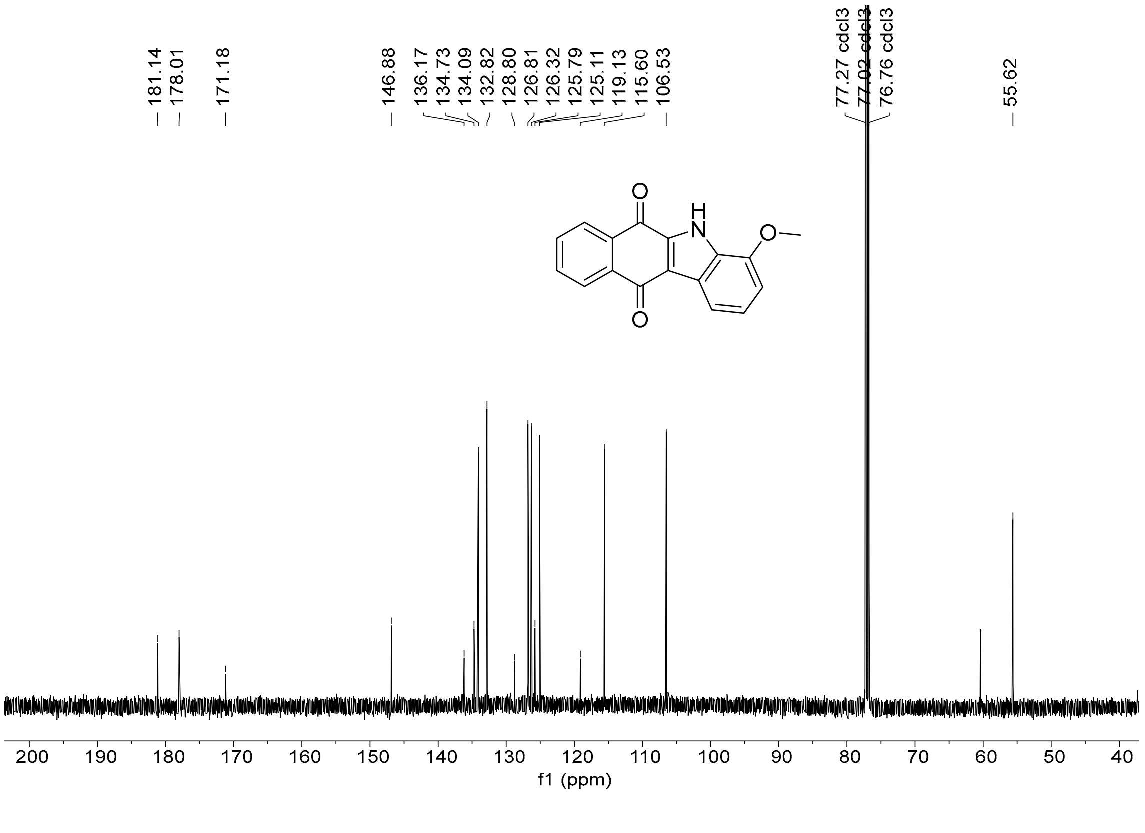

^{1}H NMR (500 MHz, $CDCl_3$) spectrum of 2,4-dimethoxy-5H-benzo[b]carbazole-6,11-dione

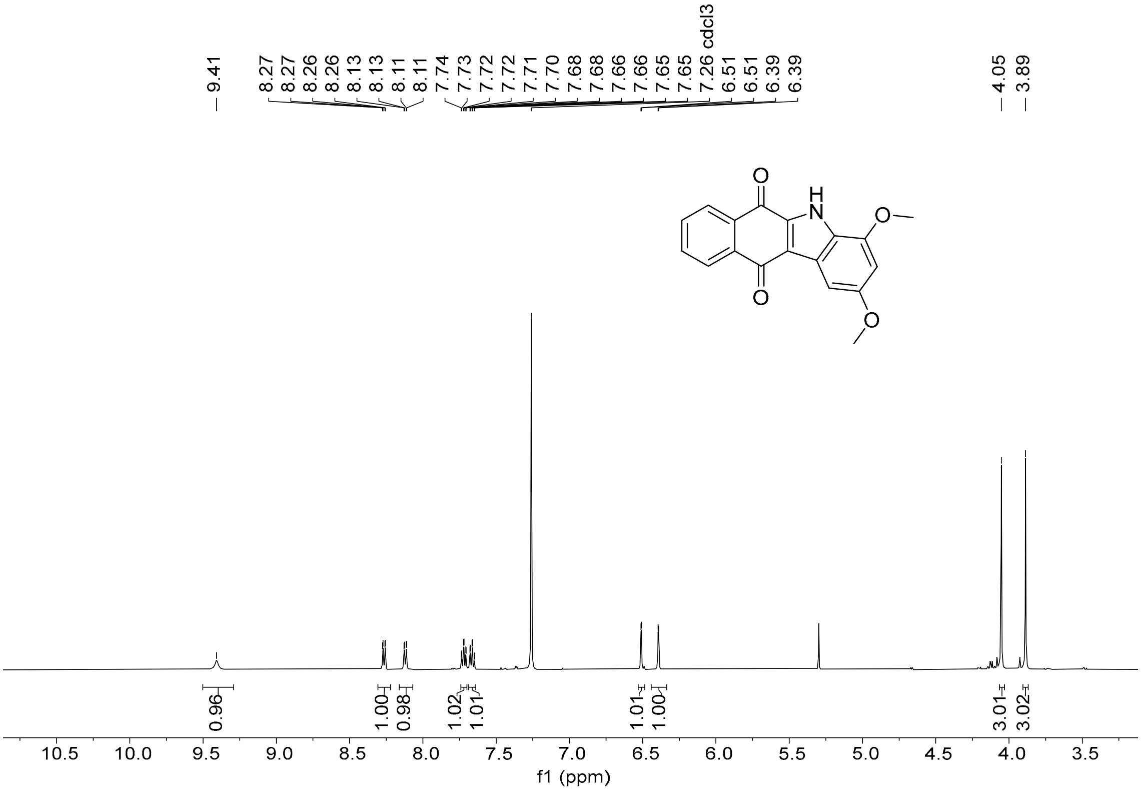

^{13}C{H} NMR (126 MHz, $CDCl_3$) spectrum of 2,4-dimethoxy-5H-benzo[b]carbazole-6,11-dione

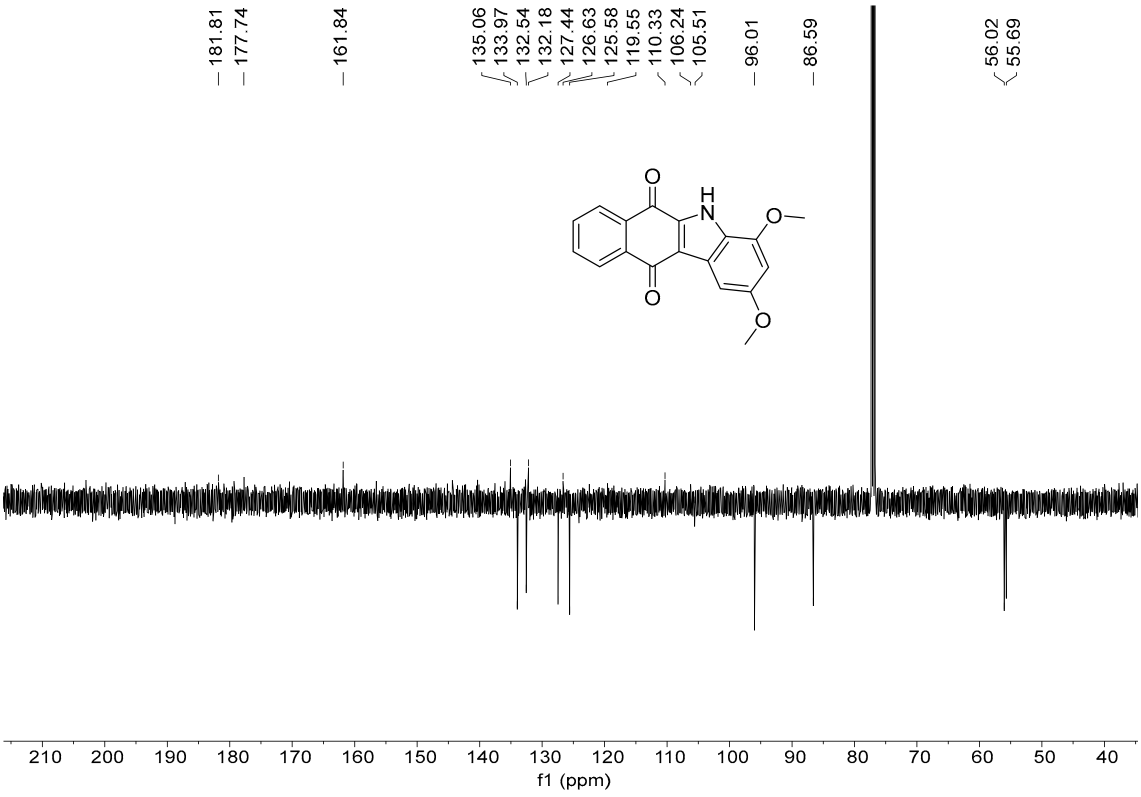

^{1}H NMR (500 MHz, $CDCl_3$) spectrum of ethyl 6,11-dioxo-6,11-dihydro-5H-benzo[b]carbazole-2-carboxylate

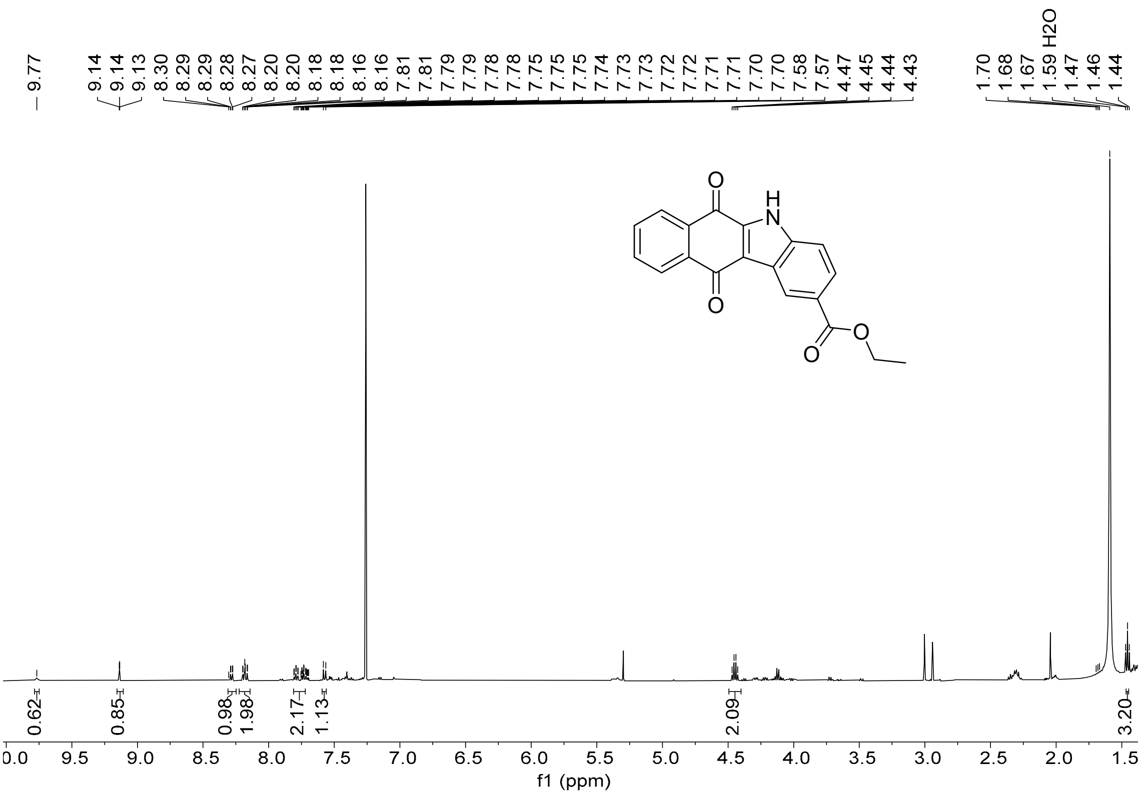

^{13}C{H} NMR (126 MHz, $CDCl_3$) spectrum of ethyl 6,11-dioxo-6,11-dihydro-5H-benzo[b]carbazole-2-carboxylate

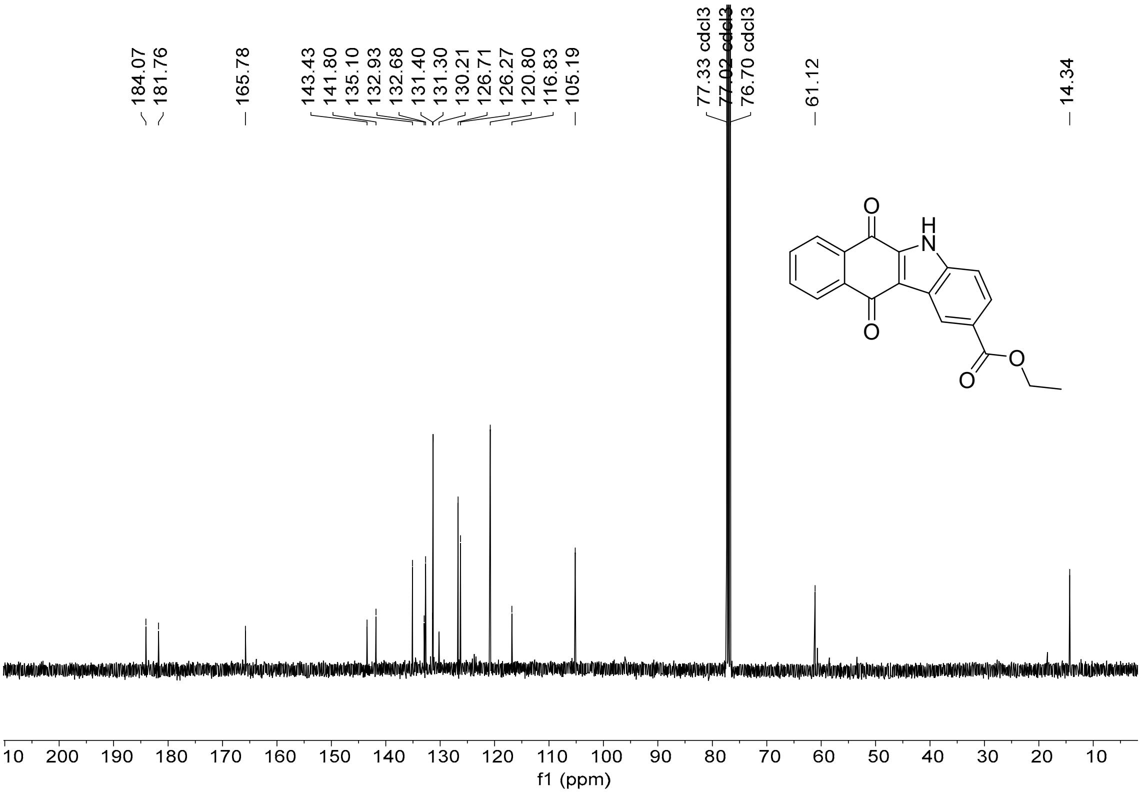

^{1}H NMR (400 MHz, $(CD_3)_2CO$) spectrum of 2-fluoro-5H-benzo[b]carbazole-6,11-dione

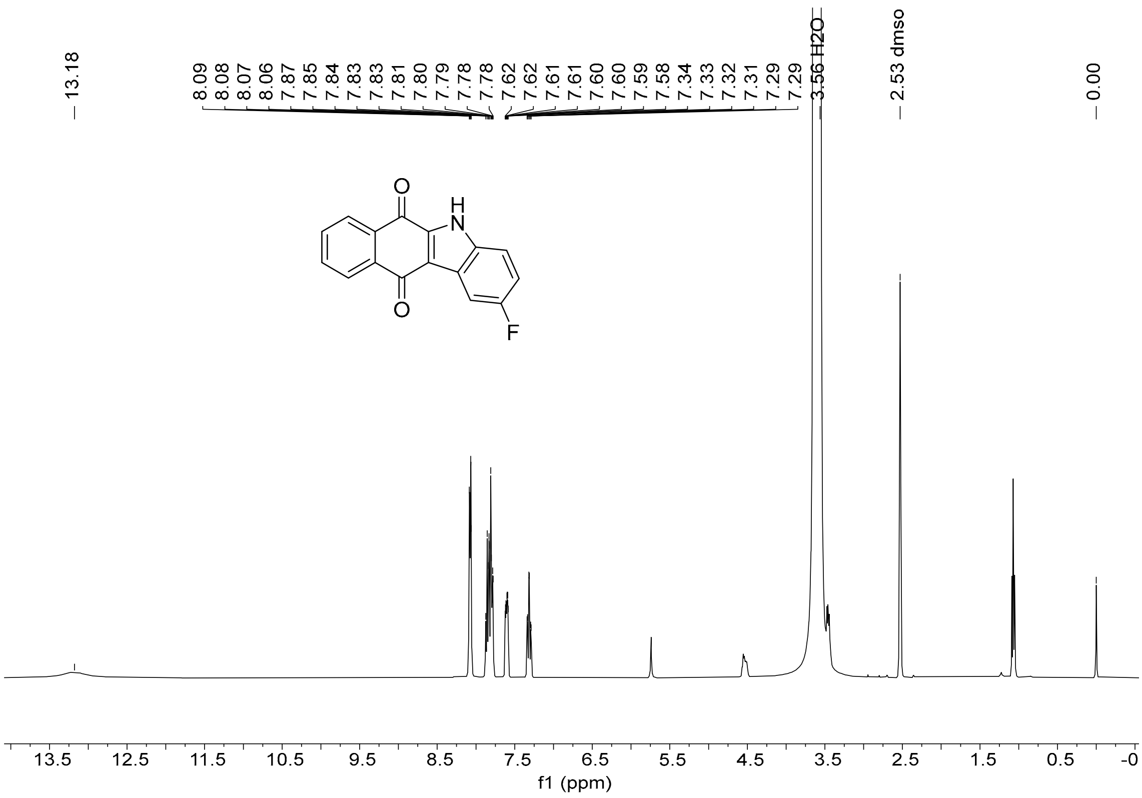

^{13}C{H} NMR (101 MHz, $(CD_3)_2CO$) spectrum of 2-fluoro-5H-benzo[b]carbazole-6,11-dione

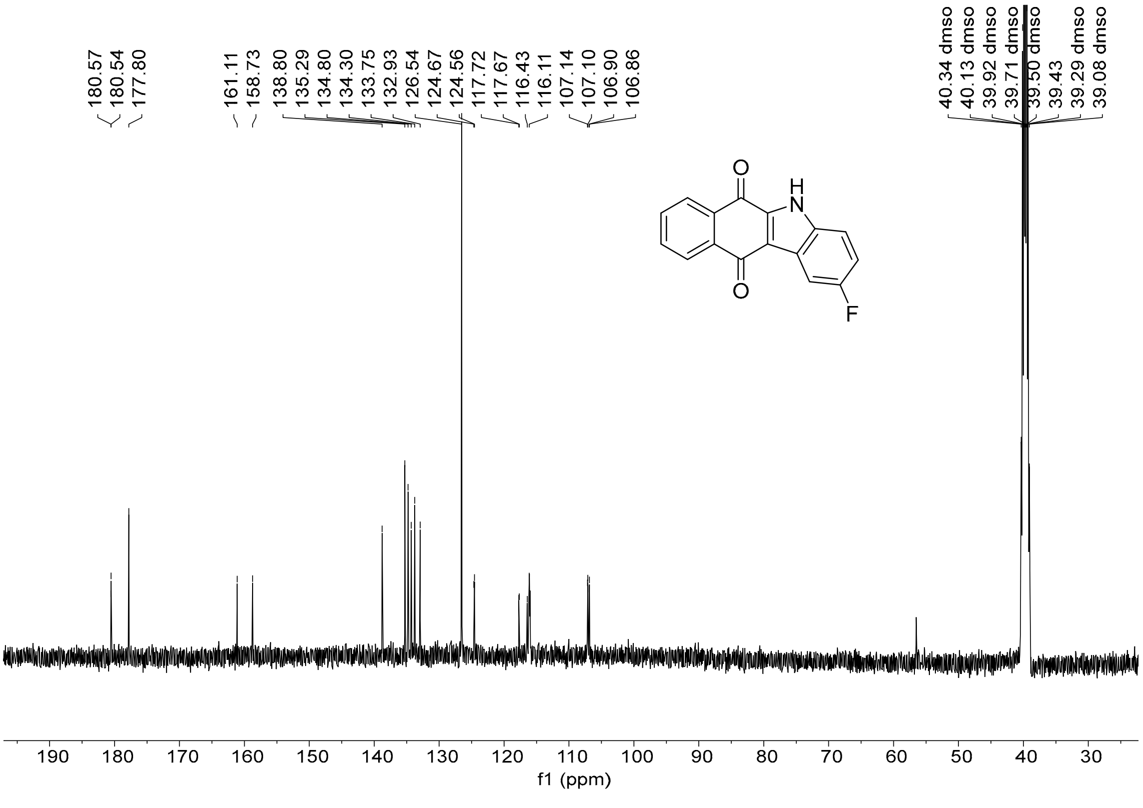

^{1}H NMR (500 MHz, $CDCl_3$) spectrum of 7H-dibenzo[a,h]carbazole-7,12(13H)-dione

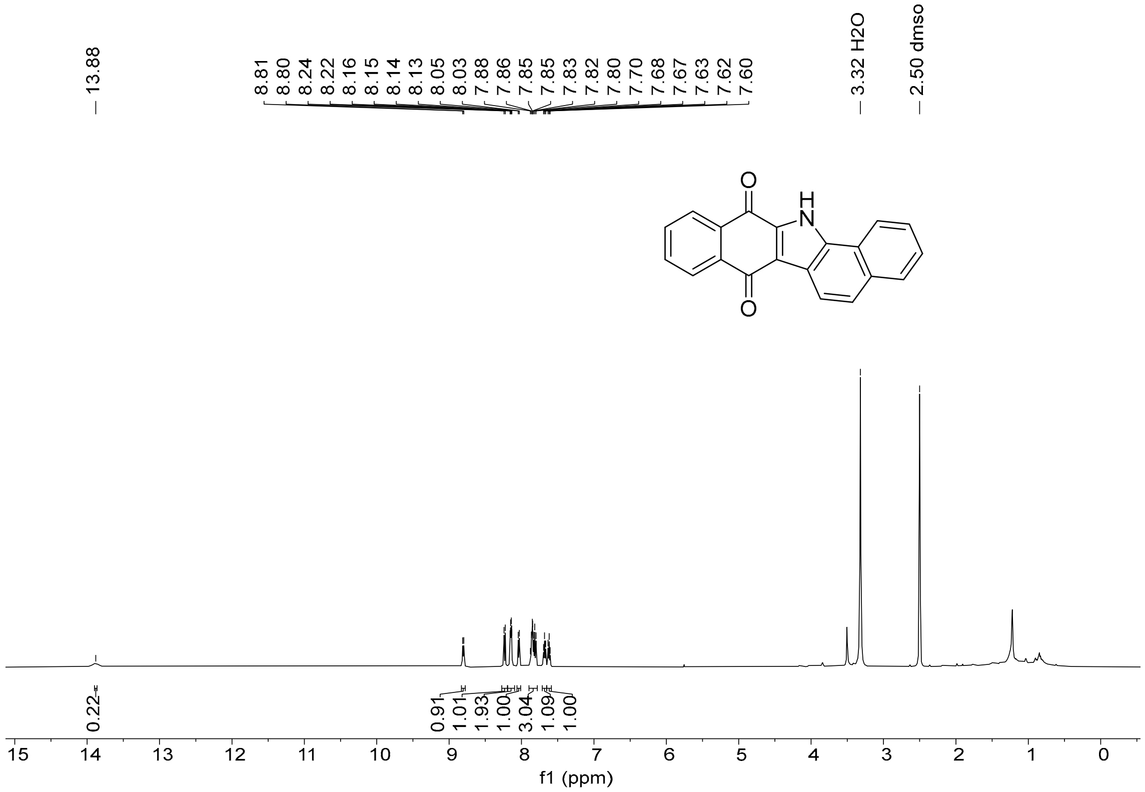

$^{13}C\{H\}$ NMR (126 MHz, $CDCl_3$) spectrum of 7H-dibenzo[a,h]carbazole-7,12(13H)-dione

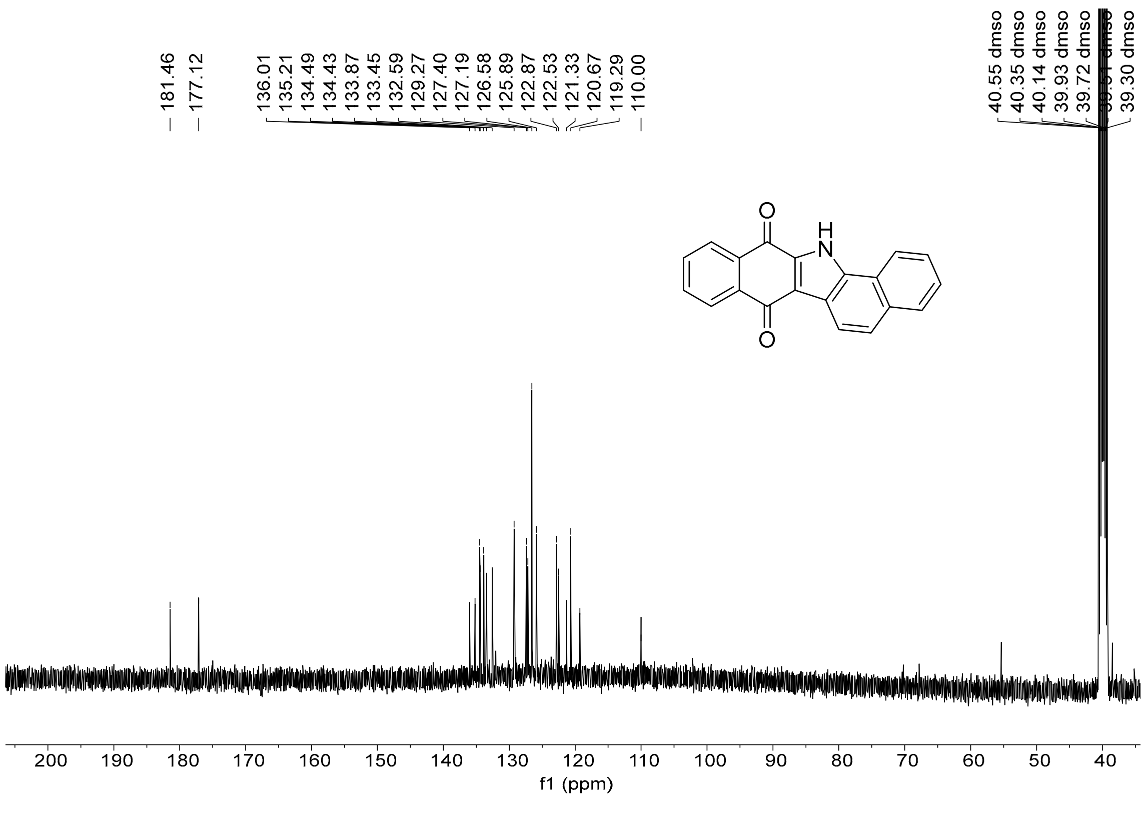

Supplement: Supplementary file 1 [file viruses-16-01768-s001.zip › viruses-3266228-supplementary.pdf]
